# Application of an Anchor Mapping of Alien Chromosome (AMAC) Fragment Localization Method in the Identification of Radish Chromosome Segments in the Progeny of Rape–Radish Interspecific Hybrids

**DOI:** 10.3390/ijms252413687

**Published:** 2024-12-21

**Authors:** Feng Zu, Xia Li, Wei Chen, Jingqiao Wang, Yanqing Luo, Sultan Mehmood, Chuchuan Fan, Jinfeng Li, Yunsong Dong, Yongming Zhou, Genze Li

**Affiliations:** 1 National Key Laboratory of Crop Genetic Improvement, Huazhong Agricultural University, Wuhan 430070, China; zufeng1984@163.com (F.Z.); fanchuchuan@mail.hzau.edu.cn (C.F.); 2 Industrial Crops Research Institute, Yunnan Academy of Agricultural Sciences, Kunming 650225, China; 18040518438@163.com (W.C.); jingqiao_wang@hotmail.com (J.W.); yanqingluo24@126.com (Y.L.); sultanmahmood195@gmail.com (S.M.); jinfengli2005@126.com (J.L.); dong_yunsong@163.com (Y.D.); 3 Biotechnology and Genetic Germplasm Resources Research Institute, Yunnan Academy of Agricultural Sciences, Kunming 650205, China; lixia_napus@163.com

**Keywords:** *Brassica napus*, *Raphanus sativus*, AMAC, alien chromosome fragments, genome-specific markers, ogura-CMS

## Abstract

Rape (*Brassica napus*) is an important oilseed crop widely cultivated worldwide. Due to its relatively short evolutionary and domestication history, its intra-species genetic diversity is limited. Radish (*Raphanus sativus*), belonging to a different genus but the same family as *B. nupus*, possesses an abundance of excellent gene resources. It is commonly used for *B. nupus* germplasm improvement and genetic basis expansion, making it one of the most important close relatives for distant hybridization. In the present study, a novel method for detecting alien chromosome fragments, called Anchor Mapping of Alien Chromosome (AMAC) was used to identify radish chromosome segments in the progeny of rape–radish interspecific hybrids. Based on the AMAC method, 126,861 pairs of IP (Intron Polymorphism) and 76,764 pairs of SSR (Simple Sequence Repeat) primers were developed using the radish Rs1.0 reference genome. A total of 44,176 markers (23,816 pairs of IP and 20,360 pairs of SSR markers) were predicted to be radish genome specific-single-locus (SSL) markers through electronic PCR analysis among four *R. sativus,* one *B. napus*, one *B. rapa,* one *B. juncea*, and one *B. juncea* reference genome. Among them, 626 randomly synthesized SSL markers (478 SSL IP markers and 148 SSL SSR markers) were used to amplify the genome of 24 radish samples (*R. sativus*), 18 rape (*B. napus*), 2 Chinese cabbage (*B. rapa*), 2 kale (*B. oleracea*), and 2 mustard (*B. juncea*) samples, respectively. Then, 333 SSL markers of the radish genome were identified, which only amplified in the radish genome and not in any *Brassica* species genome, including 192 IP markers and 141 SSR markers. Furthermore, these validated SSL markers were used to identify alien chromosome fragments in Ogura-CMS restorer line 16C, Ogura-CMS sterile line 81A, and their hybrid-Yunyouza15. In 16C, one marker, Rs1.0025823_intron_3, had an amplification product designated as anchor marker for the alien chromosome fragment of 16C. Afterwards, four novel radish genome-specific IP markers were found to be flanking the anchor marker, and it was determined that the alien chromosome segment in 16C originated from the region 8.4807–11.7798 Mb on radish chromosome R9, and it was approximately 3.2991 Mb in size. These results demonstrate that the AMAC method developed in this study is efficient, convenient, and cost-effective for identifying excellent alien chromosome fragments/genes in distant hybrid progeny, and it can be applied to the molecular marker-assisted breeding and hybrid identification of radish and *Brassica* crop species.

## 1. Introduction

*Brassica napus* (AACC, 2*n* = 38) is an important allotetraploid species within the Brassicaceae family, resulting from the hybridization of *B. rapa* (AA, 2*n* = 20) and *B. oleracea* (CC, 2*n* = 18) [1,2]. Despite being a relatively recent addition to agricultural cultivation, with an evolutionary history spanning less than 10,000 years and a commercial cultivation for oil production starting only around 700 years ago, *B. napus* has rapidly emerged as a significant global crop [2,3]. It is the second largest oilseed crop in the world, following soybean [4,5]. The genomic evolution and structural investigations of *B. napus* have indicated that its genetic diversity is very limited, and it is accompanied by a high degree of linkage imbalance in the genome, particularly in the C subgenome [3]. Therefore, a primary research focus for contemporary rapeseed researchers is the expansion of the genetic diversity of the *B. napus* gene pool and the development of more diverse and suitable germplasm materials that meet modern breeding goals such as high yield, quality, stress resistance, and mechanized harvesting. Distant hybridization among *Brassica* species is a crucial strategy for enriching and enhancing the genetic resources *of B. napus* [6]. This approach not only improves traits such as increased genetic diversity and higher yield in *B. napus* [7,8], but also broadens its genetic basis for disease resistance [9].

In addition to various species of the genus *Brassica*, which can donate superior genes/traits through distant hybridization to enhance the germplasm and gene pool of canola, other crops within the Brassicaceae family, such as *Raphanus* (radishes), *Orychophragmus*, and *Isatis*, can also act as donors through intergeneric and intertribal hybridization, providing valuable genes and traits for canola. Among these, the genus *Raphanus*, which is closely related to *Brassica*, contains genes for cytoplasmic male sterility, fertility restoration, and self-incompatibility [10]. Additionally, it possesses resistance to various diseases, i.e., root-knot nematodes, and cyst nematodes [11,12], as well as resistance against blackleg disease, clubroot, and Sclerotinia [13]. Hence, it is one of the important sources of exogenous superior genes for the improvement and enrichment of the canola gene pool. As early as 1924, Karpechenko began conducting hybridization experiments between radishes and *Brassica* to create new species or achieve gene introgression [14]. Currently, superior radish genes such as the radish cytoplasmic male sterility (CMS) type Ogura-CMS restoration gene have been introduced into canola through the distant hybridization between radish and canola, including varieties like R40 created by INRA [15], CLR650 [16], and CLR6430 [17]. Ogura-CMS is a naturally occurring cytoplasmic male sterility genome found in a radish population in Kagoshima, Japan, in 1986 [18]. This system is characterized by complete sterility and stable infertility. Paulmann and Röbbelen [19] transferred the Ogura-CMS cytoplasm into canola through intergeneric hybridization and developed the canola Ogura-CMS male sterile line through continuous backcrossing. Additionally, researchers have used the allotetraploid hybrids of canola, radish varieties, and orchid crops as a bridge, to introduce the restoration genes from orchid seed into canola, significantly improving the fertility of canola [20]. Through intergeneric hybridization, several traits such as resistance to beet cyst nematode [21], nematode resistance [12], clubroot resistance [22], and the presence of white and purple flowers [23] have been transferred to canola. This has led to the development of a large number of canola–radish monosomic addition lines [11]. Therefore, a method to quickly identify the alien chromosome fragments in the genome of these excellent *B. napus*, originating from distant hybrid offspring and providing practical molecular markers for the subsequent marker-assisted transfer of an excellent gene/trait, are worthy of research attention.

Over the past two decades, various molecular markers have been employed in genetic breeding studies of *B. napus*, including amplified fragment length polymorphisms (AFLP), simple sequence repeats (SSRs), sequence-related amplified polymorphisms (SRAP), sequence-characterized amplified regions (SCAR), and single nucleotide polymorphisms (SNP). Among these molecular markers, SSRs are considered the most suitable due to their polymorphic, co-dominant, reproducible, transferable, and abundant nature across the genome [24]. Consequently, SSRs have been widely utilized in genetic diversity studies, quantitative trait loci and genetic mapping, gene localization, germplasm classification, and evolutionary and comparative genomics. They remain one of the most important molecular markers in genetic breeding research [25]. SSR markers have been reported for the identification of heterologous chromosome fragments in interspecies/generic hybrids. A total of 22 radish genomic SSR markers linked to the Ogura-CMS restorer gene *RFO* were developed using the radish reference genome R9 chromosome sequence, and it was discovered that the chromosomal fragment carrying the restorer gene introduced into the Ogura-CMS restorer line 16C originated from the radish R9 chromosome, with a fragment size of approximately 3.30 Mb [26]. Ding et al. [27] used a set of standard materials with a known radish chromosome composition for the rape–radish addition line, and referred to the linkage map of radish C chromosome anchoring markers constructed by Nakatsuji et al. [28] and Hashida et al. [29] to establish a specific SSR marker that can accurately identify the exogenous radish C chromosome in the hybrid offspring of *B. napus* and radish C chromosomal addition line. This demonstrates that using genome-specific markers/marker bands/genotyping is a simpler and more effective method for identifying alien chromosomes/chromosome fragments in the progeny from distant hybridizations. In addition to SSR sequences, the intronic regions of genes frequently harbor a variety of repeat sequences, which play a significant role in contributing to genetic diversity. The variation across individuals on a genome-wide scale can be utilized to develop DNA markers, commonly referred to as Intron Polymorphism (IP) markers. The significance of IP markers, characterized by their co-dominant inheritance, neutrality, ease of detection, high reliability, and strong cross-transferability across related species, has led to their recognition as a novel class of molecular markers. These markers are now extensively used in various genetic analyses, including studies on rice [30,31], *Foxtail millet* [32], onion [33], carrot [34], and tea plant [35].

In the past decade, genome resequencing has been completed for various *Brassica* species, including *B*. *rapa* [36,37], *B*. *oleracea* [38,39], *B. nigra* [40], *B*. *napus* [41,42,43], *B. juncea* [44,45], and a lot of high-quality reference genome data have been obtained. With the turnip genome re-sequenced, scholars have systematically carried out collinearity comparisons and phylogenetic analyses between radish and brassica crops [46,47]. The abundant genomic information resources of *Brassica* crops are convenient for the development of specific molecular markers of the radish genome, which is closely related to it. However, there are still relatively few reports on the development and identification of heterologous chromosomes/chromosome fragments in rapeseed and radish distant hybrids, and systematic studies on identification methods are lacking. The rapid advancement of whole-genome sequencing technology has resulted in the release of the genome sequences of *B. napus* and radish, which are now available online [48,49]. These genome sequences serve as a valuable resource for the genome-wide characterization of SSR and IP. Therefore, the study employed a novel method for detecting/locating exogenous chromosome fragments/genes-Anchor Mapping of Alien Chromosome (AMAC), which was used to develop full genome-specific SSR and IP markers for radish. A radish genome-specific molecular marker map was constructed, and experimental validation was conducted using the detection of exogenous radish chromosome fragments in the known *B. napus* Ogura-CMS restorer line 16C. The research results provide theoretical and technical guidance for the identification of alien chromosome fragments in the progeny of distant hybridizations involving radish and closely related genera and species.

## 2. Results

### 2.1. Method of Anchor Mapping of Alien Chromosome (AMAC)

**Figure 1:**
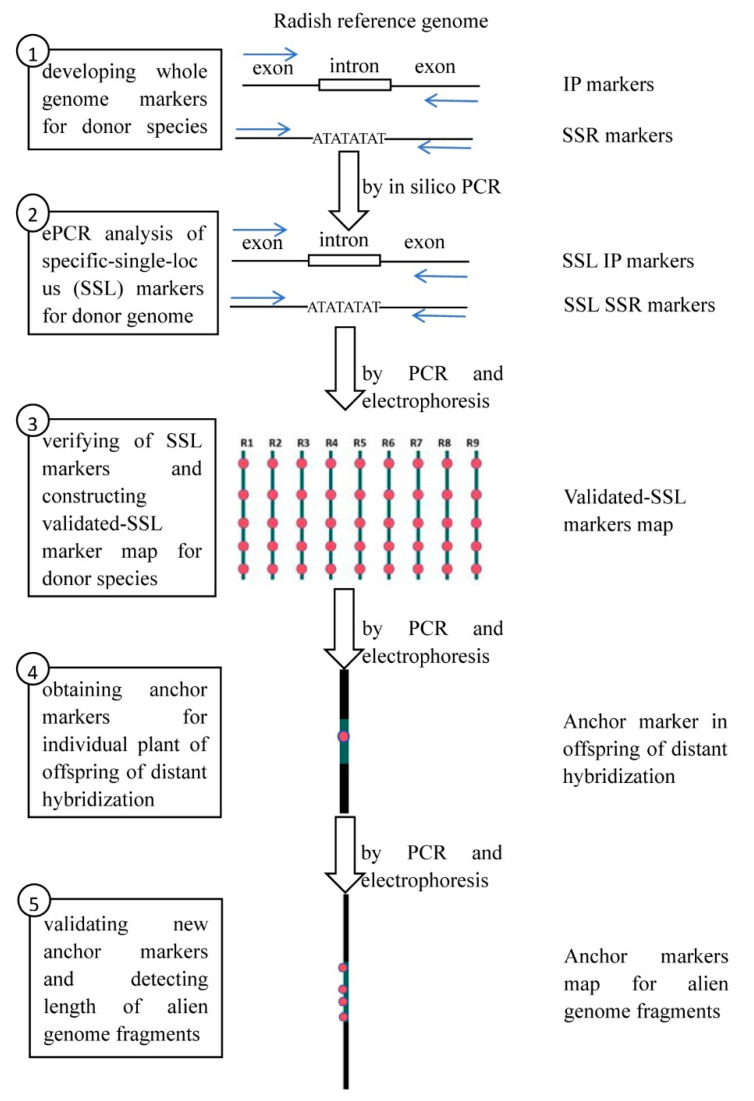
Schematic presentation of the method of anchor mapping of alien chromosome (AMAC).

The AMAC method involves five steps as follows: (1) Developing genome-wide molecular markers (SSR and IP) for the donor species of the distant hybridization. (2) Carrying out the predictive analysis of genome specific-single-locus markers (SSL markers) using in silico PCR. (3) Verifying these genome SSL markers by PCR and electrophoresis experiments, which only amplified in donor species and not in receptor species, as validated SSL markers. Then, the validated genome SSL markers are used to construct a physical map based on the markers’ position. (4) Screening validated SSL markers for alien chromosome fragments in individual offspring plant resulting from distant hybridization. (5) Further screening and validation of SSL markers upstream and downstream of the previous anchor markers for detecting the length of the alien chromosome fragments and constructing an anchor markers map (Figure 1).

### 2.2. Whole-Genome IP and SSR Marker Development and Map Construction

#### 2.2.1. Development and Mapping of Complete Genome IP and SSR Markers for Radish

**Figure 2:**
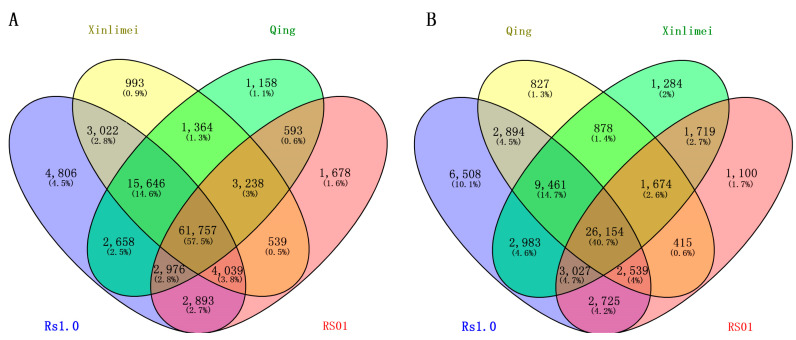
Venn diagram of IP primers (**A**) and SSR primers (**B**) expected to amplify a single locus via ePCR among the 4 radish genomes.

Using the IP_V2.0 program (2021SR0437322) developed in-house, 32,710 genes were detected in the gene annotation file of radish Rs1.0, containing a total of 162,112 introns, with an average of 4.96 introns per gene. Among these, 129,809 introns (80.1% of the total), each shorter than or equal to 200 bp, were used for the development of genome-wide IP markers in radish. Ultimately, IP primers were developed for 126,861 intron sites, accounting for 78.26% of the total number of introns. A total of 126,861 pairs of IP primers were subjected to electronic PCR analysis on the reference genomes of radish Rs1.0, Qing, Xinlimei, and RS01, respectively. The results showed that PCR amplification products were expected for 126,861, 114,108, 114,422, and 110,987 pairs of primers, respectively. Among them, the number of IP primers expected to amplify only one locus in the four radish reference genomes was 97,797, 89,390, 90,898, and 77,713, respectively (Table 1). Compared to the Rs1.0 reference genome, there were 12,753, 12,439, and 15,874 pairs of IP primers that produced no products during ePCR on the Qing, Xinlimei, and RS01 genomes, respectively, indicating that the gene loci amplified by these IP primers are either missing or mutated in these three radish genomes. Meanwhile, the number of ePCR amplification locus for 126,861 pairs of IP primers also varied among the genomes. For example, the number of IP primers expected to amplify a single locus in the RS01 genome (77,713) was significantly lower than that in the other three genomes (97,797, 89,390, and 90,598), while the number of IP primers expected to amplify multiple loci (>3) (4884) was significantly higher compared to the other three genomes (3951, 3634, and 3407) (Table 1). In the four radish genomes, the intersection among the sets of IP primers expected to amplify a single site was calculated, totaling 61,757 pairs of IP primers anticipated to amplify one site in all four radish reference genomes (Figure 2A). These IP primers were predicted to be single-locus IP markers for the radish genome.

Using MISA tool, a total of 81,213 SSR loci were identified in the radish Rs1.0 reference genome sequence, of which 76,764 loci were successfully developed into primers (Table 1). The results of electronic PCR showed that a total of 64,131 pairs of SSR primers were expected to amplify one locus in at least one of the reference genomes of Rs1.0, Qing, Xin-li-mei, and RS01. Specifically, 56,291, 44,842, 47,180, and 39,353 pairs of SSR primers were expected to amplify one locus in each of these four radish reference genomes, respectively (Table 1); 26,154 pairs of SSR primers were expected to amplify one locus in all four radish reference genomes, accounting for 40.7% of all SSR primers expected to amplify a single locus (Figure 2B).

#### 2.2.2. Analysis of Radish Genome Specific-Single-Locus IP and Single-Locus SSR Markers (Compared with Brassica Crops)

**Figure 3:**
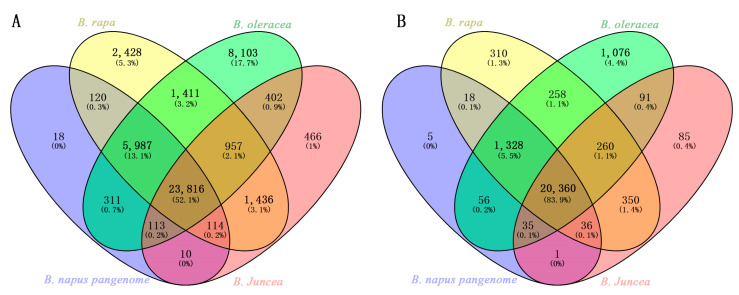
Venn diagram of IP (**A**) and SSR (**B**) markers without ePCR products in the genomes of the four *Brassica* species.

In the radish genome, 61,757 pairs of single-locus IP markers were analyzed via ePCR against the reference genomes of *Brassica*—*B. rapa*, *B. oleracea*, *B. juncea*—and the pan-genome of *B. napus*. The results indicated that a total of 37,941 radish single-locus IP markers were expected to produce PCR amplification products in at least one of the four *Brassica* species. Specifically, 25,457 pairs of primers were anticipated to amplify in the *B. rapa* genome, 20,626 pairs of primers in the *B. oleracea* genome, 34,442 pairs of primers in the *B. juncea* genome, and 31,246 pairs in the *B. napus* pan-genome (Table 2). Reverse selection, where markers were expected not to produce PCR products, resulted in 36,299, 41,130, 27,314, and 30,489 pairs of primers in the respective four *Brassica* species. Combining these reverse selection results revealed that 23,816 pairs of radish single-locus IP markers were predicted not to produce PCR products in any of the four *Brassica* crops (Figure 3A). These 23,816 are predicted to be radish genome specific-single-locus IP markers (compared to *Brassica* crops) (Table S1), with 23,518 pairs of primers expected to amplify loci distributed across the nine chromosomes (R1–R9) of the radish R genome and 298 pairs of primers located on scaffolds or contigs. On the nine chromosomes of the radish R genome, an average of 6796 single-locus IP markers and 2613 genome specific-single-locus IP markers were noted, with average marker densities of 187.84 and 72.22 markers per 1 Mb, respectively. The highest numbers and densities of both single-locus IP markers (9684 pairs, 238.52 per Mb) and specific-single-locus IP markers (3749, 92.34 per Mb) were on chromosome R5, while the lowest were on chromosome R08, with 3829 (138.73/Mb) single-locus IP markers and 1553 (56.27/Mb) genome specific-single-locus IP markers (Table 3).

A total of 25,780 single-locus SSR markers located on the nine chromosomes of the radish reference genome were analyzed using electronic PCR across the reference genomes of *Brassica*—*B. rapa*, *B. oleracea*, *B. juncea*—and the pan-genome of *B. napus*. The results indicated that 5420 radish single-locus SSR markers were expected to generate amplification products in at least one of the reference genomes among these four species. Specifically, 2860, 2316, 4562, and 3941 radish single-locus SSR markers were anticipated to have PCR products in the reference genomes of these four species, respectively (Table 2). In a reverse selection, 22,920, 23,464, 21,218, and 21,839 radish single-locus SSR markers were expected not to produce PCR products in the aforementioned *Brassica* species; taking the union of the markers without the expected amplification products revealed that 20,360 radish genome specific-single-locus SSR markers (Table S2) were expected not to amplify in the genomes of *B. rapa*, *B. oleracea*, *B. juncea*, and the pan-genome of *B. napus* (Figure 3B), and they were named radish genome specific-single-locus SSR markers (compared to *Brassica* crops). On average, each radish chromosome harbored 2864 single-locus SSR markers and 2262 genome-specific SSR markers, with an average marker density of 79.16 and 62.52 SSR markers per 1 Mb, respectively (Table 3). The R1 chromosome had the highest number of single-locus SSR markers (4143); the R8 chromosome had the lowest (1603); the R05 chromosome had the highest SSR marker density, averaging 90.27markers per Mb; the R08 chromosome had the lowest SSR marker density, averaging 58.07 markers per Mb; the R04 chromosome had the highest number of genome-specific SSR markers (3330); the R08 chromosome had the fewest genome-specific SSR markers (1300); the R05 chromosome had the highest density of genome-specific SSR markers, averaging 71.26 per Mb; the R08 chromosome had the lowest SSR marker density, averaging 47.10 per Mb (Table 3).

### 2.3. Validation of Radish Genome Specific-Single-Locus IP and SSR Marker and Map Construction

#### 2.3.1. Screening of Radish Genome Specific-Single-Locus Markers

**Figure 4:**
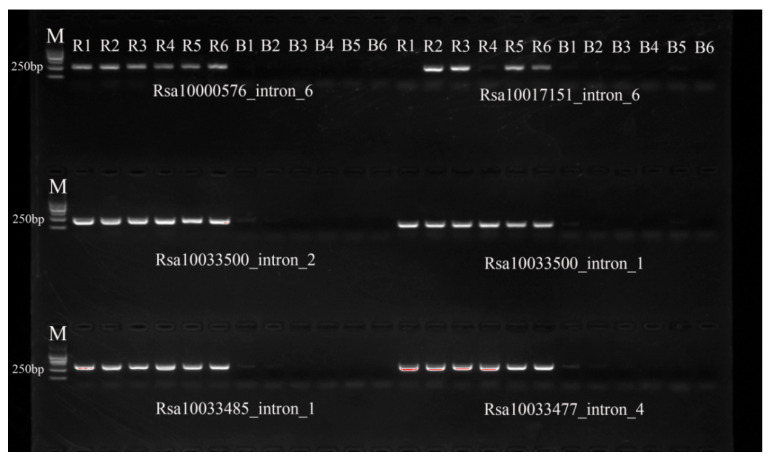
Screening of specific single-locus markers with 6 radish and 6 rapeseed accessions. M means marker; R1–R6 represent the 6 radish accessions; B1–B6 represent the 6 rapeseed accessions.

A total of 626 single-locus markers predicted by ePCR were used to amplify six radish and six rapeseed accessions to screen radish genome specific-single-locus markers. Among these, 192 pairs of IP (Table S3) markers and 141 pairs of SSR markers (Table S4) showed amplification products in four or more radish materials, while no amplification products were observed in the six hybrid rapeseed materials (Figure 4).

#### 2.3.2. Population Amplification Validation of Radish Genome Specific-Single-Locus IP and SSR Markers

**Figure 5:**
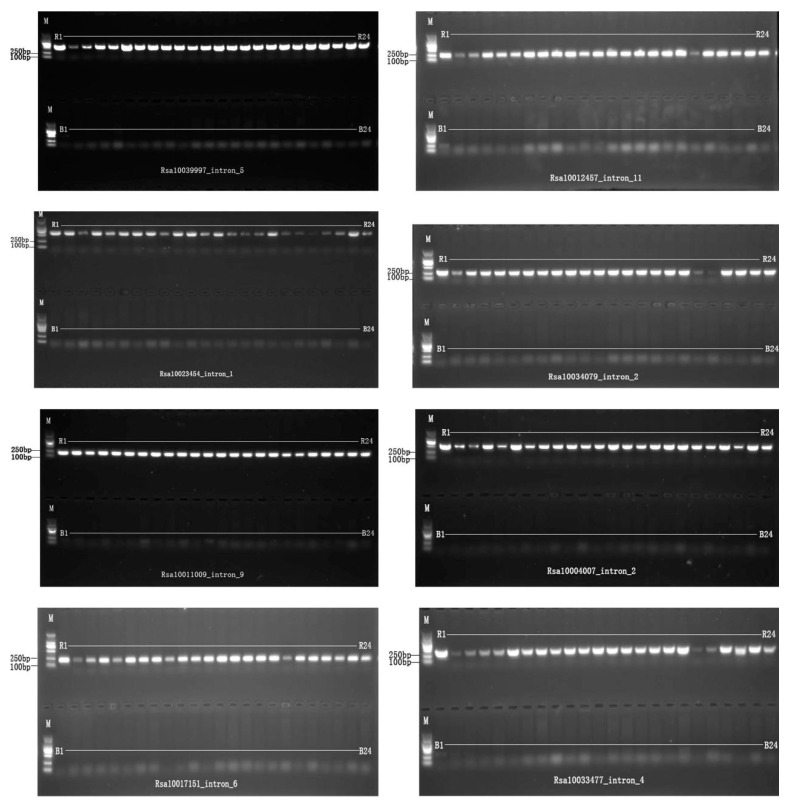
Population analysis of specific single-locus markers with 24 radish and 24 *Brassica* crops. Note: M means marker; R1–R24 represent the 24 radish accessions; B1–B24 represent the 24 *Brassica* crops.

The 192 pairs of IP markers (Table S3) and 141 pairs of SSR markers (Table S4), which exhibited amplifications specific to the radish genome, were subjected to population validation. They were used to perform PCR amplification on population samples consisting of 24 radish varieties and 24 *Brassica* varieties. The results demonstrated that all markers were validated as radish genome-specific markers. Specifically, these 192 pairs of IP markers and 141 pairs of SSR markers demonstrated amplification exclusively in radish varieties, with no amplification products observed in the 24 *Brassica* varieties (Figure 5).

#### 2.3.3. Radish Genome Specific-Single-Locus IP and SSR Marker Map Construction

The distribution of the 333 validated radish genome specific-single-locus markers on radish chromosomes is not uniform (Table 4). The R09 chromosome has the highest coverage density with 36 single-locus specific markers, averaging 1.31 Mb per specific marker. The lowest coverage density is found on the R03 chromosome, with only 23 single-locus specific markers, averaging 0.82 Mb per specific marker. On average, there is one specific marker every1.02 Mb across the entire R genome (Table 4, Figure S1).

### 2.4. Detecting Exogenous Chromosomal Segments in the Ogura-CMS Restoration Line 16C of B. napus Based on the Method of AMAC

#### 2.4.1. Analysis of Radish Genome-Specific Markers in Ogura-CMS Restoration Line 16C

**Figure 6:**
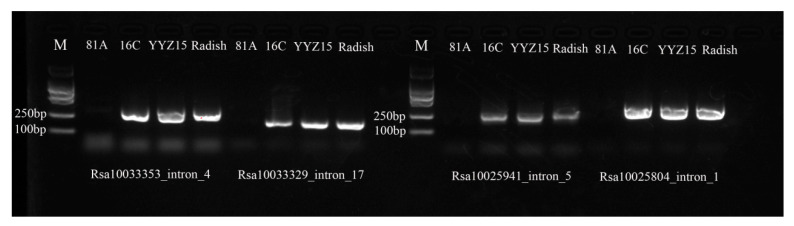
Specific IP marker linkage with *RFO* gene in 16C. M means marker; 81A means Ogura-CMS male sterile line; 16C means Ogura-CMS male sterile restorer line; Yunyouza15 is derived from the hybrid of 16C × 81A.

**Figure 7:**
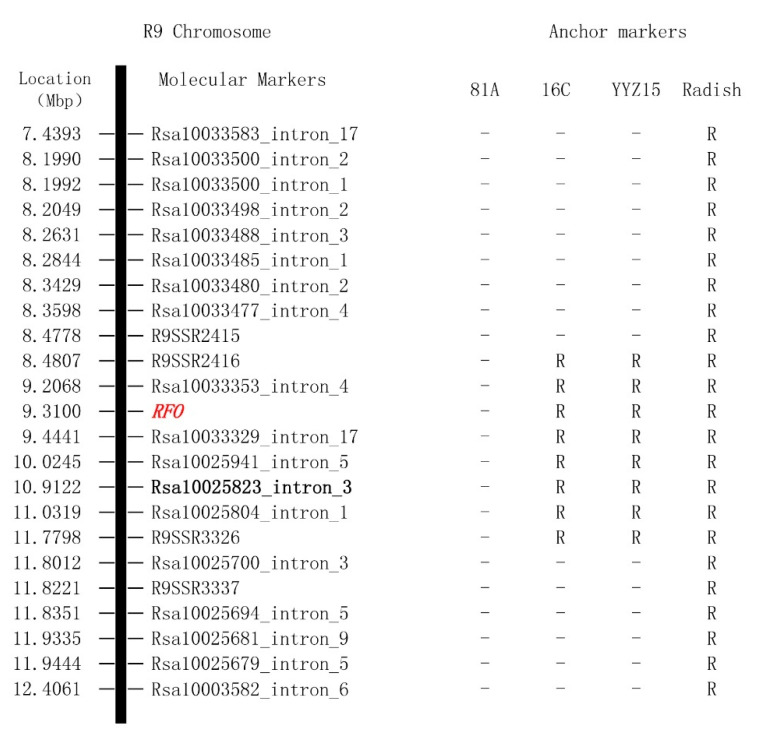
Radish genome specific markers located in the chromosome of Ogura-CMS restoration line 16C. Note: 16C means Ogura-CMS restoration line 16C, 81A means Ogura-CMS male sterile line, Yunyouza15 is derived from the hybrid of 16C × 81A, RFO means Ogura-CMS restore gene, words in bold show the specific anchor marker, R represents a radish fragment, and –represents absence of a radish fragment.

A total of 333 radish genome specific-single-locus markers (Figure S1, Tables S3 and S4) were used to amplify the genome of the hybrid line Yunyouza 15, Ogura-CMS restoration line 16C, and Ogura-CMS sterile line 81A (Figure 6) with PCR. It was demonstrated that the single-locus IP marker Rs1.0025823_intron_3 yielded amplification products in Yunyouza 15 and Ogura-CMS restoration line 16C, while no amplification products were observed in Ogura-CMS sterile line 81A (Figure 6). This marker was identified as an anchor marker for the exogenous radish chromosomal segment, located within the interval of chromosome R09 from 10912208 to 10912415 (Figure 6, Table 5) and linked with gene *RFO* (Figure 7). Using the Rs1.0025823_intron_3 marker as the anchor point, four new radish genome specific-single-locus IP markers (Rs1.0033353_intron_4, Rs1.0033329_intron_17, Rs1.0025941_intron_5, and Rs1.0025804_intron_1) were discovered to be flanking it (Table 5). All these markers showed amplification products in Ogura-CMS restoration line 16C and Yunyouza 15 but not in Ogura-CMS sterile line81A (Figure 6).

#### 2.4.2. Specific Marker Map of Exogenous Radish Chromosomal Segment in Ogura-CMS Restoration Line 16

To further clarify the interval of the radish chromosomal segment containing the exogenous restoration gene in the Ogura-CMS restoration line 16C, this study also conducted PCR analysis on four pairs of SSR markers, namely R9SSR2415, R9SSR2416, R9SSR3326, and R9SSR3337, which have been previously reported to be linked to the *RFO* gene [27]. These markers were analyzed in 24 radish varieties and 24 *Brassica* species. Among them, R9SSR2415 and R9SSR3326 are reported as the two terminal markers of the exogenous chromosomal segment in Ogura-CMS restoration line 16C, while R9SSR2416 and R9SSR3337 are the non-differential markers closest to R9SSR2415 and R9SSR3326, respectively [26]. The results showed that these four SSR markers are also radish genome-specific molecular markers.

Combining the results of previous studies with those of this study, a radish genome-specific marker map of the exogenous radish chromosomal segment carried by the restoration gene in 16C was constructed (Figure 7). R9SSR2416 and R9SSR3326 still remain as the two terminal markers of this exogenous chromosomal segment. R9SSR2415 remains the closest non-differential radish-specific marker to R9SSR2416. Rs1.0025700_intron_3 replaces R9SSR3337 as the new non-differential radish-specific marker closest to R9SSR3326. The exogenous chromosomal segment in Ogura-CMS restoration line 16C originates from the region of the radish R9 genome spanning 8.4807–11.7798 Mb, which is approximately 3.2991 Mb in size. The predicted boundaries of the ends of the imported exogenous segment are estimated to be approximately 2.9 Kb between 8.4778 and 8.4807 Mb and approximately 21.4 Kb between 11.7798 and 11.8012 Mb.

## 3. Discussion

### 3.1. Batch Development of IP Markers at the Genome Level in Plants

Species identification, population genomics, and molecular ecology studies depend on the availability of highly polymorphic independent markers. Introns are non-coding regions of DNA located within the genes of all eukaryotic genomes, exhibiting a significant level of variability in terms of length and sequence composition, which makes them suitable candidates for multi-allelic DNA markers [30,50]. Among simple PCR-based markers, IP markers demonstrate gene specificity, high variability, environmental neutrality, and co-dominance, resulting in a high transferability rate across related species [51]. The advancement of whole-genome sequencing, combined with the availability of robust in silico tools, can facilitate the development of low-cost, highly efficient gene-associated functional molecular markers for genotyping [52]. Although IP markers have been widely utilized in genetic mapping, population relationship analysis, and comparative studies among closely related species across various crops [35,50,53], no reports have addressed methods for developing batch IP markers that cater to the needs of “dummy-style” operations for ordinary users, along with statistical summaries of ePCR analysis results. Therefore, this study provides a convenient method, IP_V2.0 (2021SR0437322), for the batch development of IP primers for reference species’ whole genomes and ePCR analysis. IP_V2.0 is an interactive running program with simple operation, requiring only the input of five parameters as prompted by the user to achieve the rapid batch development of IP markers for reference species’ whole genomes and statistical analysis of ePCR. Additionally, IP_V2.0 contains four subprograms, each capable of independent operation, to meet the users’ personalized analysis needs. Moreover, ePCR enables the rapid computer analysis of primer amplification sites expected in the genome, suitable for pre-analysis before primer selection, and has been widely applied in SSR marker evaluation analysis of crops such as *B. napus* [54], *Kaempferia parviflora* [55], and coconut [56]. In this study, using this program, the development and ePCR analysis of 126,861 pairs of IP primers for the radish genome (with Rs1.0 as the reference genome) were completed within 4 h, greatly improving the efficiency of IP marker development and analysis.

### 3.2. The AMAC Method for the Rapid and Accurate Identification of Exogenous Chromosome Fragments in Distant Hybrid Offspring Lines

Distant hybridization is an important technique for promoting species improvement and evolution, enabling the transfer and exchange of large chromosomal segments and desirable genes between species, and it is widely used in the modern genetic breeding of various crops [57,58,59]. Accurately and quickly identifying exogenous chromosome fragments and genes in the genomes of offspring from distant hybridized superior lines presents significant challenges. Genomic in situ hybridization (GISH) and fluorescence in situ hybridization (FISH) are traditional cell biology techniques commonly used to detect chromosomal variations, and they have been widely applied in identifying exogenous chromosomes or chromosome fragments in *B. napus* and radish distant hybrid offspring [16,22]. However, GISH and FISH techniques are cumbersome, requiring high technical expertise, specialized experimental platforms, lengthy detection cycles, and substantial costs, rendering them unsuitable for the large-scale detection of distant hybrid offspring [6,59]. Additionally, their detection results can only prove the existence of exogenous chromosomes, making it difficult to resolve the size and boundary sites of these exogenous chromosome fragments and limiting their further application in modern biological breeding [6,59]. The AMAC method proposed in this study has the advantages of simple operation, low technical thresholds and experimental platform requirements, short cycles, and low costs. Based on this method, we constructed a radish genome-specific marker map consisting of 215 single-locus markers specific to the radish genome, with an average of one specific marker per 2.08 Mb. Using this radish genome-specific marker map, five specific marker sites were detected in the Ogura-CMS restoration line 16C, integrating previous research results [26], and further confirming that the exogenous chromosome segment in 16C originates from the region of the radish R9 genome corresponding to the 8.4807–11.7798 Mb interval, approximately 3.2991 Mb in size. The boundaries of the exogenous imported fragment on both sides were determined to be approximately 2.9 Kb between 8.4778 and 8.4807 Mb and approximately 21.4 Kb between 11.7798 and 11.8012 Mb. These results demonstrate the feasibility and efficiency of the ECAL method in identifying and analyzing exogenous chromosome fragments in the *B. napus* genome.

### 3.3. The Importance of Radish Genomic-Specific Molecular Markers in Assisting the Breeding of Ogura CMS Restorer Lines

Rapeseed is one of the crops for which heterosis utilization is quite common. Among the two sterility systems currently employed in rapeseed, CMS is favored due to its complete sterility, low cost, and high profitability, in contrast to genic male sterility (GMS), which is characterized by incomplete sterility and higher costs. The Ogura-CMS system, initially discovered in Japanese radish, is characterized by the complete anther abortion that remains unaffected by environmental conditions. Its fertility restoration gene, *RFO*, is exclusively present in radish, making almost all *B. napus* lines maintainers of this gene. After introducing the sterility-inducing and restoration genes from radish into *B. napus* via distant hybridization/cell fusion, the Ogura-CMS system has become one of the most successful commercially utilized systems for harnessing hybrid vigor in *B. napus*, widely applied in breeding hybrid varieties in Europe and America. However, Ogura-CMS also comes with drawbacks such as poor nectary development affecting insect pollination, chlorosis, and difficulty in finding suitable restoration sources. Moreover, when introducing the restoration gene into *B. napus*, many undesirable traits such as high glucosinolate and erucic acid content are inevitably brought in. Previous research has shown that most high glucosinolate genes are tightly linked to the Ogura-CMS restoration gene *Rfo* [6,16,59,60,61,62,63,64]. Therefore, indiscriminately reducing glucosinolate content may result in the loss of partial restoration gene fragments, which can lead to a decreased or complete loss of the restoration capability of Ogura-CMS. Since only the restoration gene *Rfo* is required for the Ogu-CMS pollination system, theoretically, the shorter the exogenous radish chromosome segments in the restoration line, i.e., the less redundant radish genetic information is linked with the restoration gene *Rfo*, the better the genetic stability and agronomic traits of the restorer line. Therefore, the development of radish-specific markers linked to *Rfo* is crucial for enhancing the selection of *B. napus* restorer lines with shorter exogenous restoration gene chromosomal segments through mutagenesis and the subsequent selfing or backcrossing generations. The Ogura-CMS restorer line 16C used in this study is also a superior double-low restorer line selected from distant hybrid offspring of radish and *B. napus*, and its male parent, Yunyouza 15, was selected as a recommended dominant grain and oil variety by the Ministry of Agriculture and Rural Affairs in 2022. This study developed five new radish genome-specific single nucleotide IP molecular markers closely linked to the *Rfo* gene. Preliminary research results included the construction of a linkage map containing 4 pairs of SSR markers and 18 pairs of IP molecular markers, significantly increasing the number of markers closely linked to the *Rfo* gene, which helps break the patent restrictions on *Rfo* gene-linked molecular markers [26]. Furthermore, the study results further determined that the exogenous chromosomal segment in 16C originates from the segment corresponding to the radish R9 genome region of 8.4807–11.7798 Mb, approximately 3.2991 Mb in size. The predicted boundaries of the exogenous insertion fragment were approximately 2.9 Kb between 8.4778 and 8.4807 Mb and approximately 21.4 Kb between 11.7798 and 11.8012 Mb, different in size and origin from the exogenous chromosomal segment carried by the European Ogura CMS restorer line R2000, providing data support for further investigation into the specific location of the exogenous chromosomal insertion into the *B. napus* genome and the development of co-dominant molecular markers.

## 4. Materials and Methods

### 4.1. Plant Materials and DNA Extraction

In order to formulate genome-specific molecular markers for the radish genome, the study included 24 diverse genotypes from *Brassica* crops providing a negative control (Table 6), while 24 genotypes from radish germplasm were used as positive controls (Table 7). Furthermore, the hybrid rapeseed variety Yunyouza 15 (*RFOrfo*) and its parental materials 16C (*RFORFO*) and 81A (*rforfo*), selected as test genotypes for detecting and mapping alien chromosomal fragments incorporated into Brassica crops carrying the restorer gene RFO, were provided by the rapeseed center of the Economic Crops Research Institute of the Yunnan Academy of Agricultural Sciences. The DNA extraction was conducted using the well-established CTAB extraction method [27]. The integrity and quality of the extracted DNA were subsequently assessed using 1.0% agarose gel electrophoresis.

### 4.2. Genome Sequence

The genome sequences of radish Rs1.0 were downloaded from the Radish Genome Database website, which was used to develop whole-genome IP and SSR markers for radish. The genome sequences of radish and *Brassica* used for the IP and SSR molecular marker electron PCR analysis were as follows: the genome sequences of *B. rapa* (Brara_Chiifu_V3.0) [35], *B. oleracea* (Braol_JZS_V1.1) [65], and *B. juncea* (Braju_tum_V1.5) [44] were downloaded from the website http://brassicadb.org (accessed on 7 January 2022), the pan-genome sequences of *B. napus* was downloaded from the website http://cbi.hzau.edu.cn/bnapus (accessed on 10 September 2020) [42], the genome sequences of Qing were downloaded from https://ftp.ncbi.nlm.nih.gov/genomes/all/GCA/902/824/885/GCA_902824885.1_Qing (accessed on 25 September 2021), and the genome sequences of Xin-li-mei and RS01 were downloaded from https://ngdc.cncb.ac.cn/bioproject/browse/PRJCA003033 (accessed on 13 July 2020) [66].

### 4.3. Development and Identification of Radish Genome-Specific IP and SSR Markers Based on Anchor Mapping of Alien Chromosomal Fragment (AMAC)

#### 4.3.1. Development of Whole-Genome IP and SSR Markers Based on Radish Genome and Electronic PCR Analysis

The development of IP markers based on the radish genome employs the IPv2.0 program, which was independently developed by the Industrial Crops Research Institute, Yunnan Academy of Agricultural Sciences. This software is registered under copyright number 2021SR0437322. The process involves importing the annotation file and corresponding genome sequence file into the server, along with specifying the upper limit of intron length. Subsequently, the IPv2.0pl script is executed to retrieve intron information, including gene location, length, and sequence. The radish genome sequence information file is then uploaded, and the IP primer design sequence is generated using the IP_p3in. v2.0.pl script, using the intron information obtained in the previous step. Finally, the result file generated by the IP_p3out.v2.0.pl script is used to design IP primers using Primer3.0. To further refine primer selection and maximize the utility of the IP primers, the designed IP primers were screened using the ePCR method in silico [67] with the following parameters: 3 bp mismatch, 2 bp gap, 60 bp margin, and 80–200 bp product size. The target IP markers are subsequently screened based on the results of this ePCR analysis.

The MISA tool was employed to filter SSR regions with the following default parameter settings: unit_size and min_repeats set as 1-12 2-6 3-5 4-5 5-5 6-5, and interruptions (max_difference_between_2_SSRs) set as 100 [68].

Subsequently, short oligonucleotide primers were designed for the filtered IP and SSR regions using Primer 3.0, following the standard primer design protocols as follows: the primer length was maintained between 18 and 24 bp, with an optimal size of 22 bp; the melting temperature ranged from 55 °C to 65 °C, with an optimal temperature of 60 °C; the GC content was between 40% and 60%; and the predicted PCR product size was between 80 and 450 bp. All other parameters were set to their default values.

#### 4.3.2. Prediction of Single-Locus Markers and Genome Specific-Single-Locus Markers in Radish

Based on the electronic PCR results of the whole-genome IP and SSR primers developed for radish genomes, the IP and SSR markers predicted to amplify a single locus in all four radish reference genomes are designated as radish genome single-locus IP markers and radish genome single-locus SSR markers, respectively. These radish single-locus IP and SSR markers are then subjected to electronic PCR analysis in the genomes of *Brassica* crops—including *B. rapa*, *B. oleracea*, *B. juncea*—and the pan-genome of *B. napus* (with the same parameter settings as Section 4.3.1). The electronic PCR results are employed in a reverse selection process, in which any radish single-locus IP and single-locus SSR markers anticipated to amplify in any of the reference genomes of *B. rapa*, *B. oleracea*, *B. juncea*, and the pan-genome of *B. napus* are excluded. Ultimately, only those markers that consistently amplify a single locus in all four radish genomes and are not expected to yield PCR products in any of the reference genomes of *B. rapa*, *B. oleracea*, *B. juncea*, and the pan-genome of *B. napus* are selected. These are predicted to be radish genome specific-single-locus IP markers or radish genome specific-single-locus SSR markers (as compared to *Brassica* crops).

#### 4.3.3. Validation of Radish Genome Specific-Single-Locus IP and SSR Marker

To validate the actual specificity of the predicted radish genome specific-single-locus IP or genome specific-single-locus SSR markers, based on the expected amplification information from the ePCR analysis, a total of 478 pairs of IP primers and 148 pairs of SSR primers were synthesized. Using these synthesized molecular markers, PCR amplification was conducted on materials from 24 radish varieties (Table 7) and 24 *Brassica* crop materials (12 hybrids of *B. napus*, 6 conventional types of *B. napus*, 2 types of *B. juncea*, 2 types of *B. rapa*, and 2 types of *B. olereace*) (Table 6), randomly selecting 6 samples from each (R1–R6 and B1–B6). Markers that amplified in four or more radish materials but not in any *Brassica* samples were selected. Using the selected markers, PCR amplification was carried out on the 24 radish samples and 24 *Brassica* crop populations to confirm them as radish genome-specific molecular markers.

### 4.4. Application of AMAC Method in Detecting Exogenous Chromosomal Segments in Ogura-CMS Restoration Line 16C of B. napus

Radish genome specific-single-locus IP and SSR markers validated in the method shown in Section 4.3.3 were used to amplify the genome of the hybrid line Yunyouza 15, Ogura-CMS restoration line 16C, and Ogura-CMS sterile line 81A. If a marker showed amplification products in Yunyouza 15 and Ogura-CMS restoration line 16C but not in Ogura-CMS sterile line 81A, it was considered as an anchor marker for the exogenous radish chromosomal segment. Markers flanking the anchor marker were then selected and validated by PCR amplification on populations of radish and *Brassica* species using single-locus IP and SSR markers specific to the radish genome (see Section 4.3.3 for details). Radish genome-specific markers validated by population were subsequently used for the PCR amplification in Yunyouza 15 of *B. napus*, Ogura-CMS restoration line 16C, and Ogura-CMS sterile line 81A; the interval of the exogenous radish chromosomal segment in the Ogura-CMS restoration line 16C of *B. napus* was determined through construction of a marker map. The online tool for drawing genetic maps, MG2C_V2.1 (http://mg2c.iask.in/mg2c_v2.1/index.html, accessed on 3 March 2022) [69], was used to construct the marker map with the default parameters. The positions of the markers were determined based on the results of the ePCR analysis using the reference genome sequence.

## 5. Conclusions

In the present study, we developed 203,625 primer pairs using radish Rs1.0 as the reference genome, including 126,861 pairs of IP primers and 76,764 pairs of SSR primers, among which 44,176 pairs were specific to the radish genome. A radish genome-specific marker map was constructed using 333 validated radish genome-specific molecular markers (compared to Brassicaceae crops), with an average of one radish genome-specific marker per 1.02 Mb. Based on five radish genome specific-single-locus IP markers, one anchor marker and four flanking markers, this study determined that the exogenous chromosomal segment in 16C originated from the segment corresponding to the region of 8.4807–11.7798 Mb on the radish R9 chromosome and has an approximate size of 3.2991 Mb. These results confirm the effectiveness and feasibility of the AMAC method, not only in detecting the presence of chromosomal segments, but also in mapping alien chromosomal segments.

## Figures and Tables

**Table 1:** Statistical results of ePCR analysis of 126,861 IP and 76,764 SSR pairs of primers in the 4 radish genomes.

Ref. Genome	ePCR 1 Locus No.IP (No.SSR)	ePCR 2 Loci No.IP (No.SSR)	ePCR 3 Loci No.IP (No.SSR)	More than 3 Loci No.IP (No.SSR)	Total No.IP (No.SSR)
Rs1.0	97,797 (56,291)	20,038 (8413)	5075 (2669)	3951 (9391)	126,861 (76,764)
Qing	89,390 (44,842)	16,935 (6068)	4149 (1879)	3634 (7158)	114,108 (59,947)
Xinlimei	90,598 (47,180)	16,752 (3890)	3665 (1426)	3407 (6011)	114,422 (58,507)
RS01	77,713 (39,353)	22,353 (7106)	6037 (1688)	4884 (6427)	110,987 (54,574)

**Table 2:** Statistical results of ePCR analysis with 61,757 single-locus IP markers and 25,780 single-locus SSR markers in the 4 *Brassica* species genomes.

Ref. Genome	ePCR 1 Locus No.IP (No.SSR)	ePCR 2 Loci No.IP (No.SSR)	ePCR3 Loci No.IP (No.SSR)	More than 3 Loci No.IP (No.SSR)	Total No.IP (No.SSR)
*B.rapa*	22,728 (2690)	2424 (146)	226 (16)	79 (8)	25,457 (2860)
*B.oleracea*	18,334 (2179)	2015 (116)	216 (16)	61 (5)	20,626 (2316)
*B.juncea*	14,113 (2644)	13,599 (1390)	4364 (382)	2366 (146)	34,442 (4562)
*B.napus*	6538 (1356)	10,602 (1426)	6650 (661)	7477 (498)	31,267 (3941)

**Table 3:** Distribution of single-locus IP (SSR) and genome specific-single-locus IP (SSR) in the radish genome.

Chromosome	Total Length (Mb)	Count of Single-Locus IP (SSR)	Density of Single-Locus IP (SSR)	Count of Genome Specific-Single-Locus IP (SSR)	Density of Genome Specific-Single-Locus IP (SSR)
R01	51.80	9394 (4143)	181.35 (79.98)	3614 (3275)	69.77 (63.22)
R02	40.50	7954 (3393)	196.40 (83.78)	3051 (2688)	75.33 (66.37)
R03	27.90	5113 (2176))	183.26 (77.99)	1972 (1718)	70.68 (61.58)
R04	49.80	9416 (4192)	189.08 (84.18)	3740 (3330)	75.10 (66.87)
R05	40.60	9684 (3665)	238.52 (90.27)	3749 (2893)	92.34 (71.26)
R06	26.20	4604 (1845)	175.73 (70.42)	1739 (1451)	66.37 (55.38)
R07	26.00	4948 (2014)	190.31 (77.46)	1791 (1557)	68.88 (59.88)
R08	27.60	3829 (1603)	138.73 (58.08)	1553 (1300)	56.27 (47.10)
R09	35.20	6226 (2748)	176.88 (78.07)	2309 (2148)	65.60 (61.02)
Average	36.18	6796 (2864)	187.84 (79.16)	2613 (2262)	72.22(62.52)
Total	325.60	61,168(25,780)	187.86 (79.18)	23,518 (20,360)	72.23 (62.53)

**Table 4:** Distribution of specific single-locus markers validated by PCR in radish genomes.

Chromosome	Total Length (Mb)	Counts of Specific Single-Locus Markers	Density of Specific Single-Locus Markers
R01	51.80	50	0.97
R02	40.50	42	1.04
R03	27.90	23	0.82
R04	49.80	46	0.92
R05	40.60	37	0.91
R06	26.20	29	1.10
R07	26.00	29	1.12
R08	27.60	31	1.12
R09	35.20	46	1.31
Average	36.18	37	1.02
Total	325.60	333	1.02

**Table 5:** Information of the 4 novel specific single-locus IP markers linked with the *RFO* gene.

Marker ID	Forward Primer 5′-3′	Reverse Primer 5′-3′	Chromosome	Start	End
Rs1.0033353_intron_4	AACACATGCGAATCACTGGA	AAGGGACGGTCAAGGAACTT	R09	9,206,762	9,206,971
Rs1.0033329_intron_17	GCTCTCGGAAGCTGTTGTGT	CTCCCAAAACCTCGCTGTAG	R09	9,444,064	9,444,238
Rs1.0025941_intron_5	CCCAGGAAGCAAAGTATGGA	CAGAGGCACAGCTGAAATTG	R09	10,024,454	10,024,660
Rs1.0025823_intron_3	CCAAGGAGGGCTCTTCAACT	CGTGCTAATGGGTTTTGGAT	R09	10,912,208	10,912,415
Rs1.0025804_intron_1	CAAAACCAGGGAGAAGGACA	GCAACGAGAAGCCTTTCATC	R09	11,031,906	11,032,298

**Table 6:** List of *Brassica* crops used in this study.

Brassica Crops	Serial Number	Varieties	Soure
*B. napus* hybrid variety	B1	Chuanyou45	Sichuan
	B2	Chuanyou81	Sichuan
	B3	Chuanyou83	Sichuan
	B4	Liangyou100	Sichuan
	B5	Dexinyou198	Sichuan
	B6	Dechaoyou797	Sichuan
	B7	Demingyou700	Sichuan
	B8	Xingaoyou478	Sichuan
	B9	Huaza62	Hubei
	B10	Fuyou2	Fujian
	B11	Baoyouza2	Yunnan
	B12	Baoyouza3	Yunnan
*B. napus* conventional variety	B13	Yunyouhuazaoshu1	Yunnan
	B14	Yunyoushuang2	Yunnan
	B15	Huayou6	Yunnan
	B16	Huayou8	Yunnan
	B17	Huauyou9	Yunnan
	B18	Yuhongyou4	Yunnan
*B. juncea* variety	B19	Baoshanhuangyoucai	Yunnan
	B20	Fengyiliyuancaizi	Yunnan
*B. oleracea* variety	B21	Korea Chunxiao	Korea
	B22	Weifengniuxin	Yunnan
*B. rapa* variety	B23	Chinese cabbage	Yunnan
	B24	Wutacai	Jiangsu

**Table 7:** Information of the 24 radish varieties used in this study.

No. Sample	Varieties	Leaf Shape	Root Shape	Root Color	Fleshy Color
R1	Tianjinshawo	Flower leaf	Spindle	Blue and white	Green
R2	French Breakfast	Flower leaf	Long ellipse	Water red and white	White
R3	Purple Plum	Flower leaf	Cone	Purple	White
R4	Easter Egg	Flower leaf	Rotundity	Red, white, purple, or rose	White
R5	Spanish Black	Flower leaf	Flat rotundity	Black or black-purple	White
R6	White Hail Cherry	Flat leaf	Flat rotundity	White	White
R7	Pioneer Cherry	Flower leaf	Rotundity	Water red	White
R8	Medium Red Cherry	Flower leaf	Rotundity	Water red	White
R9	Italian Black Long	Flower leaf	Spindle	Black or black-purple	White
R10	Dutch Roulade Cherry	Flower leaf	Rotundity	Red	White
R11	South Korean Summer White Jade	Flat leaf	Long cylinder	White	White
R12	Busan Spring Snow	Flower leaf	Spindle	White	White
R13	Dutch Yellow Cherry	Flat leaf	Long ellipse	Yellow	White
R14	White Cherry	Flower leaf	Flat rotundity	White	White
R15	Jiujindawang	Flower leaf	Long cylinder	White	White
R16	Xinlimei (Beijing)	Flower leaf	ellipse	Blue and white	Rose red
R17	Sanchibai	Flower leaf	Long cylinder	White	White
R18	Xinlinmei	Flower leaf	ellipse	Blue	Purplish red
R19	Ice Cream	Flower leaf	ellipse	Blue	White powder alternating
R20	Ziyutang Fruit	Flower leaf	Spindle	Purple	White and purple alternating
R21	ZIdan	Flower leaf	Cylinder	Purple	White and purple alternating
R22	Pineapple Fruit	Flower leaf	Spindle	Blue	Light green
R23	Flower Cherry	Flower leaf	Long cone	Light pink and white	White
R24	Weixian	Flower leaf	Spindle	Blue	Green

## Data Availability

All data are shown in Tables and Figures in the main text or in the Supplemental Materials.

## Supplementary Materials

The following supporting information can be downloaded at: https://www.mdpi.com/article/10.3390/ijms252413687/s1.
