# Supplementary figures and images for: Application of an Anchor Mapping of Alien Chromosome (AMAC) Fragment Localization Method in the Identification of Radish Chromosome Segments in the Progeny of Rape–Radish Interspecific Hybrids
Source: Int J Mol Sci. 2024 Dec 21;25(24):13687. doi: 10.3390/ijms252413687 (PMC11728025; doi:10.3390/ijms252413687)

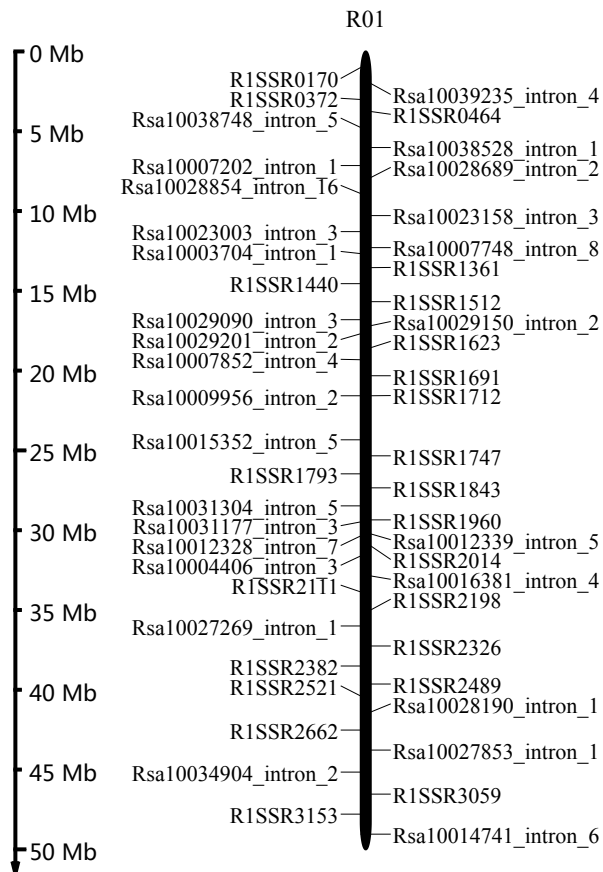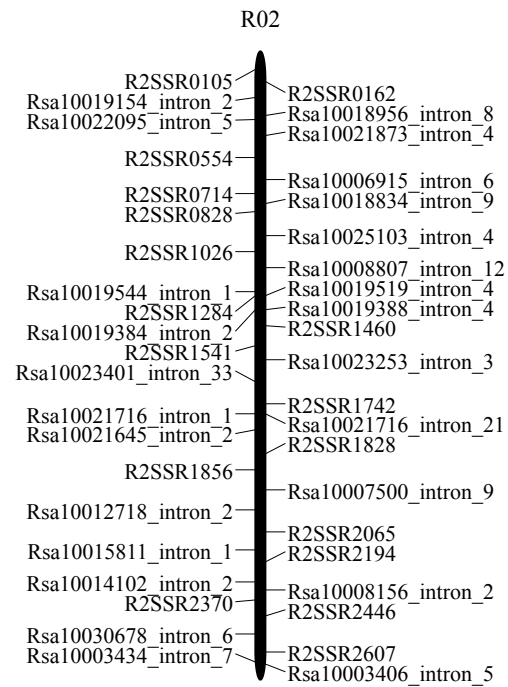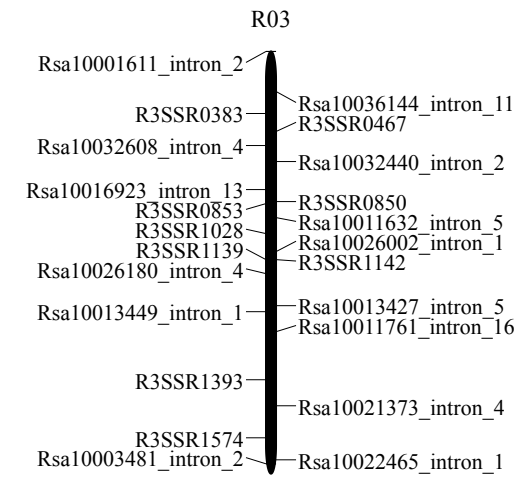

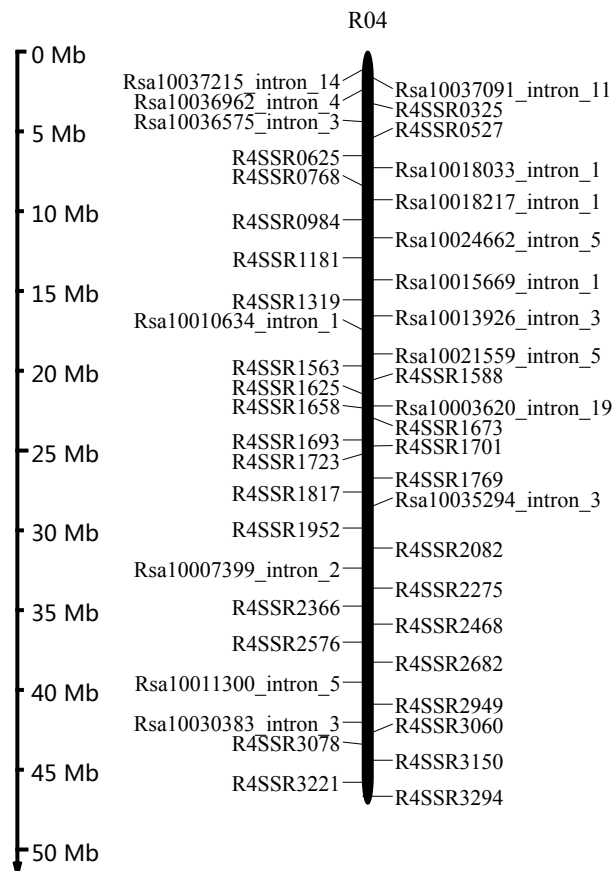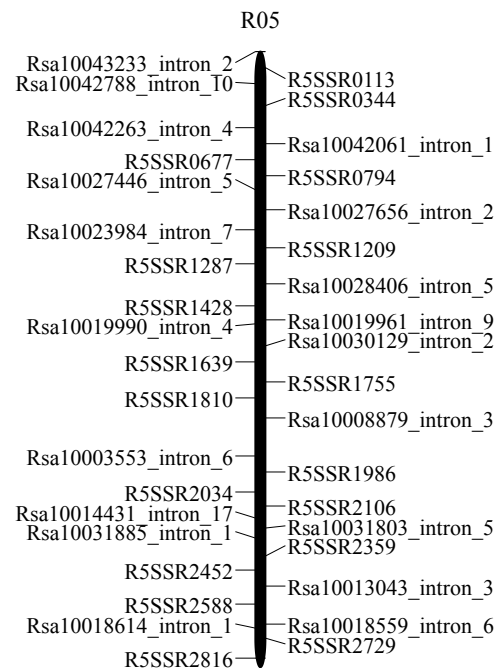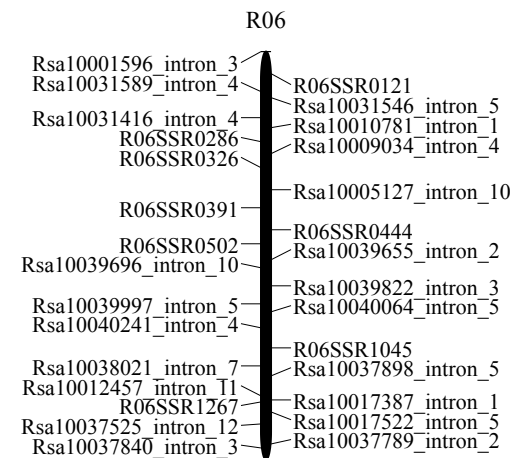

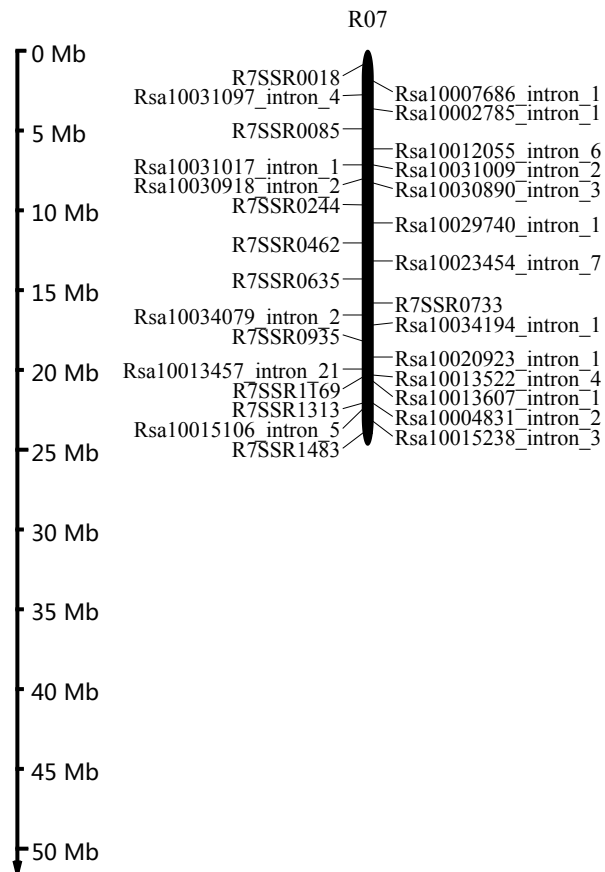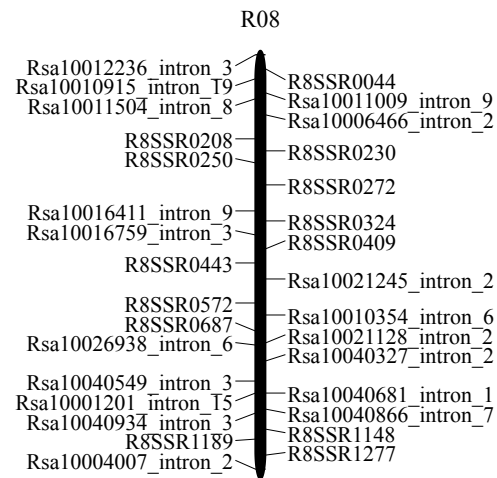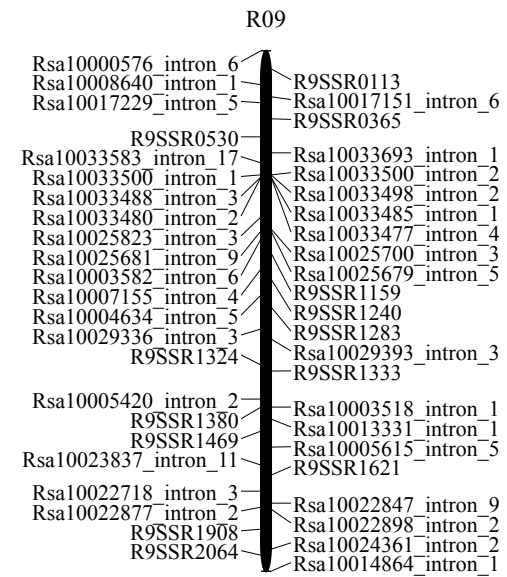

Supplement: Supplementary file 1 [file ijms-25-13687-s001.zip › Figure S1. Map of 333 radish genome specific single-locus markers validated by PCR. The left of map shows location in Radish genome; the right shows the single-locus markers..pdf]
